# From In-Person to the Online World: Insights Into Organizing Events in Bioinformatics

**DOI:** 10.3389/fbinf.2021.711463

**Published:** 2021-09-07

**Authors:** Alessandra Lima da Silva, Ana Paula de Abreu, Diego Mariano, Felipe Caixeta, Fenícia Brito Santos, Fernanda Stussi D. Lage, Gabriel Quintanilha-Peixoto, Heron. O. Hilário, Joicymara. S. Xavier, Lucio. R. Queiroz, Nayara Evelin de Toledo, Raphael Tavares, Rodrigo Bentes Kato, Roselane Gonçalves dos Santos, Stellamaris Soares, Wanessa. M. Goes, Wylerson. G. Nogueira, Thiago. M. Batista, José Miguel Ortega, Vasco Ariston Azevedo De Carvalho, Glória. Regina Franco, Raquel. C. de Melo-Minardi, Aristóteles Góes-Neto

**Affiliations:** ^1^ Institute of Biological Sciences, Universidade Federal de Minas Gerais (UFMG), Belo Horizonte, Brazil; ^2^ Department of Clinical and Toxicological Analysis, Faculty of Pharmacy, Universidade Federal de Minas Gerais, Belo Horizonte, Brazil; ^3^ Department of Computer Science, Universidade Federal de Minas Gerais (UFMG), Belo Horizonte, Brazil; ^4^ Genomics for Climate Change Research Center, Universidade Estadual de Campinas (Unicamp), Campinas, Brazil; ^5^ Conservation Genetics Laboratory, Pontificia Universidade Catolica de Minas Gerais (PUC Minas), Belo Horizonte, Brazil; ^6^ Rene Rachou Institute (Fiocruz Minas), Belo Horizonte, Brazil; ^7^ Institute of Agricultural Sciences, Universidade Federal dos Vales do Jequitinhonha e Mucuri (UFVJM), Unaí, Brazil; ^8^ Environmental Science Training Center, Universidade Federal do Sul da Bahia, Porto Seguro

**Keywords:** Bioinformatics, computational biology, education, Science popularization, online events

## Abstract

Bioinformatics is a fast-evolving research field, requiring effective educational initiatives to bring computational knowledge to Life Sciences. Since 2017, an organizing committee composed of graduate students and postdoctoral researchers from the Universidade Federal de Minas Gerais (Brazil) promotes a week-long event named Summer Course in Bioinformatics (CVBioinfo). This event aims to diffuse bioinformatic principles, news, and methods mainly focused on audiences of undergraduate students. Furthermore, as the advent of the COVID-19 global pandemic has precluded in-person events, we offered the event in online mode, using free video transmission platforms. Herein, we present and discuss the insights obtained from promoting the Online Workshop in Bioinformatics (WOB) organized in November 2020, comparing it to our experience in previous in-person editions of the same event.

## Introduction

Education in bioinformatics is a transdisciplinary practice involving extensive knowledge in biology, statistics, mathematics, and data science (LIU et al., 2013). The demand for qualified professionals in this area emerged with the advent of big data, which in biological sciences, corresponds to topics such as DNA and RNA sequencing data, and studies of protein structure (NCBI, 1988; Illumina, 2021). Consequently, integrative educational initiatives in bioinformatics are necessary to introduce and develop the required skills. Such initiatives could also help fulfill the demands of both academia and industry concerning research progress and the creation of new tools, products, and technologies (Wilson Sayres et al., 2017; Rocha, 2021). In Brazil, disseminating the theoretical and practical knowledge in bioinformatics is mainly led by academics linked to public institutions. There are seven registered graduate programs in bioinformatics and one in computational biology, besides a technological course in bioinformatics, which make up the education and research network in this country. Moreover, other biological sciences courses (e.g., undergraduate courses in biotechnology, biology, and graduate courses in genetics) usually include disciplines related to bioinformatics.

Graduate students play a key role in the teaching of bioinformatics. Some graduate programs and scholarships criteria require students to enroll in teacher training, which is also relevant to science popularization (CAPES, 2021). Disciplines in undergraduate and graduate courses, winter/summer schools and courses, workshops, seminars, and short courses are some of the most common event formats for science popularization in bioinformatics (Ranganathan, 2005; Welch et al., 2014; Atwood et al., 2015). In these formats, lecturers with different levels of training and expertise communicate their knowledge to heterogeneous audiences, contemplating students in universities, technical courses, professionals in the field, and even the university staff (Fuentes-Acosta et al., 2021). The covered subjects might fit into the most diverse segments of bioinformatics, ranging from basic concepts of molecular biology and computer science to Omics, MetaOmics, and Structural Biology (Gauthier et al., 2019).

During 2020, in-person events and classes were unfeasible due to the COVID-19 pandemic. Therefore, universities and students had to adapt to online classes (Mahmood, 2021). Nonetheless, many students do not possess the setup required for online classes, including a quiet room to study, reliable access to the internet, or even a compatible device (computer, tablet, mobile, and others). In addition, many students share a computer with their whole family. Interactive classes try to overcome this challenge by keeping the student concentrated on the computer screen for longer (Ibrahem and Alamro, 2020). Additionally, not seeing the audience (at least on some free streaming platforms) is another challenge for the speakers. During in-person events, speakers stand up in front of their audience and use non-verbal communication, such as reactions and facial expressions to improve their talk (Wood-Harper, 2021). On the other hand, providing an online event avoids many costs, such as travel, food, and accommodation, providing a free and globally accessible meeting that can attract a larger number of attendees (Reshef et al., 2020), despite the quality of learning, which may be inferior.

Considering this scenario, in this work, we bring the experience of the first Online Workshop in Bioinformatics (WOB) compared to our latest in-person event, the Summer Course in Bioinformatics (CVBioinfo). We prioritized engagement through quizzes and surveys during lectures, awards, and other actions to help bring the attendees together. Moreover, other activities, from posts on social media to emails with relevant topics about the online event and short breaks during the event, had a relevant impact. We also prioritized accessibility in WOB, so that all the attendees could participate and use the content without any restriction (at least 12.7 million people in Brazil have some disability or limitation) (IBGE, 2010). This report hopes to provide a model for the science popularization of online events in bioinformatics and to reflect on possible improvements through the feedback of our attendees.

## Materials and Methods

The attendees were required to fill an application form for participating in both CVBioinfo and WOB. These forms were available for approximately 1 month, comprising quantitative and qualitative sections collecting primary data (e.g., name, age, email address, and so on) and interest data from a list of topics (only for WOB), including computer science (e.g., programming languages, operational systems, and machine learning), omics sciences, and transdisciplinary topics (e.g., ecology, science outreach, and entrepreneurship). In previous CVBioinfo editions, we also required candidates to provide a resumé (generated in the Lattes platform, which is the standardized repository of curricula of Brazilian researchers) and a motivational letter, but this was not required in the last edition (nor did we levy any participation fees). Those surveys allowed not only to gather the data for this study but also guided the speakers of the audience’s expertise concerning their presentation topic. In addition, we sent a feedback form to the attendees, enquiring about the overall organization of the course, its content quality and quantity, their satisfaction and willingness to indicate it for a friend and to attend future editions, their satisfaction with the course organization, and suggestions for next editions. For WOB, especially, these forms included an opinion section for the round tables and lectures. We later correlated this data using the Orange Data Mining tool (Demsar et al., 2013).

### The Experience of the Summer Course in Bioinformatics in Previous Editions

The Summer Course in Bioinformatics (CVBioinfo) was initially designed to be a showcase of the Graduate Program in Bioinformatics of the Universidade Federal de Minas Gerais (PPGBioinfo-UFMG) and the research possibilities for undergraduates, and then attract new graduate students to the program. The Summer Course in Bioinformatics gradually evolved to interact with a broader audience, as a transdisciplinary bridge between attendees of diverse backgrounds and research in bioinformatics.

CVBioinfo was created in 2017 by graduate students and postdoctoral fellows of the PPGBioinfo-UFMG, supported by the professors and researchers of this graduate program and the university structure. The first edition comprised a diverse range of lectures in omics sciences and structural biology, establishing a successful model for the forthcoming years. Without a participation fee, this first edition had 600 attendees, mainly from UFMG and other institutions in the southeast of Brazil. Besides, seven practical mini-courses were offered on topics such as introduction to programming languages, data visualization, metagenomics, and molecular modeling. This event was an excellent opportunity for non-specialists to grasp the diversity of bioinformatics applications, gathering interest in this area.

In 2018, a symbolic registration fee was introduced to avoid the high evasion rate of the first edition, in which many attendees did not take part in the entire event. The fee was also a way to gain financial independence to plan the subsequent editions. This edition comprised 5 days of lectures and two different short practical courses per day. The number of attendees was now limited to 200 people, selected through a brief motivational letter. The Organizing Committee decided to limit the number of attendees to better serve the attendees and improve their experience, in addition to adapting the event to the best infrastructure available. The number of applications was 22% higher in the following edition (2019). That year, subscriptions came from more than 70 different universities (including five institutions in other countries) and high school students. Despite this diversity, most of the attendees were undergraduate students from the state of Minas Gerais, in Biological Sciences. In contrast to the underrepresentation of women in STEM fields (Bonham et al., 2017), female participation was 60% in this edition, as computational biology is considered an interdisciplinary STEM field.

### Structure of In-Person Editions

Following the aforementioned model, the order of activities was maintained flexible, aiming to maximize attendance and engagement. The mornings of the first day of the event were reserved for an opening lecture, in which the CVBioinfo history and the PPGBioinfo-UFMG structure were presented to attendees by the graduate program coordinator. From 2017–2019, the lectures occurred in the mornings while the afternoons were reserved for theoretical/practical courses. The lectures were 50 min long, with 10 min reserved for Q and A. In the 2020 edition, the courses happened during the mornings and the lectures in the afternoon to stimulate higher attendance rates. Speakers included professors, postdoctoral fellows, and graduate students of UFMG (not limited to any particular program or expertise). Although external speakers were always welcome, their invitation was dependent on the budget of that event. Most of the lectures introduced bioinformatics research applications and theory to the audience, using accessible language whenever possible while highlighting the interplay with other knowledge domains. The main guiding subjects were DNA, RNA, and proteins studies, as well as related technologies and techniques.

Some lectures, mainly in 2020, were dedicated to showing research lines open to new students and were conducted by PPGBioinfo-UFMG professors, while other lectures were reserved for the sponsors of the event to showcase their products and initiatives (always related to applied bioinformatics and biotechnology). Furthermore, some lectures were also in the roundtable format. Three to five speakers were invited to present short introductions on a common topic, followed by a discussion open to the public. The closing lecture was reserved for new researchers/professors that are part of the PPGBioinfo-UFMG alumni to provide an example of the paths traveled from the start of the formation as a bioinformatician to the many viable mature careers.

The Organizing Committee was supported by volunteers (only during event days), and they were indispensable for the proper functioning of the event. These volunteers aided in the space organization, reception of the attendees, and help on minor issues throughout the event. In 2020’s in-person event, the whole organization team was composed of 29 people, divided into 14 students and post-doctoral researchers from PPGBioinfo-UFMG as the main organizers of the event, plus 15 graduate students and undergraduates volunteers and the PPGBioinfo-UFMG staff (responsible for activities directly related to the budget). The main organizers were divided into six teams with different responsibilities, such as advertisement, speakers contact, website development, registration management, content creation, and interaction with attendees, encouraging them to clarify their doubts about topics covered at the event. Additionally, short coffee breaks separated the lectures. These intervals, intended for resting, were also crucial for networking, creating opportunities for the attendees to meet each other and the lecturers to extend the discussion on the presented topics, ask spare questions, and exchange contacts. Every edition also had a dinner night before the last day to complement these breaks, where attendees, speakers, and the organizing committee interacted over drinks and regional food.

### Structure of the Online Edition

A total of 20 speakers (13 from Brazilian institutions and seven from foreign institutions) were involved. The Brazilian institutions with which our speakers were associated are located in Minas Gerais (Welch et al., 2014), Bahia (NCBI, 1988), São Paulo (NCBI, 1988), and Rio de Janeiro (LIU et al., 2013), while the Brazilian speakers abroad were located in Australia (NCBI, 1988), the United States of America (NCBI, 1988), Germany (LIU et al., 2013), and England (LIU et al., 2013). For this reason, all lectures were taught in Portuguese. Eleven of these institutions are public, and eight, private. The Organizing Committee of the online edition consisted of 17 students and researchers from PPGBioinfo-UFMG (one M.Sc., 12 Ph.D. students, and four postdoctoral researchers). The organizers were divided into teams, similar to the in-person event. In addition, another team supported live lectures with live translation to Brazilian sign language (LIBRAS) from an accessibility and inclusion core at UFMG, which offers this service free of charge.

The selection of the speakers was based on the knowledge and relevance of their subject, the ability to prepare and deliver interesting short talks, which are essential in online events. Besides, our speakers were highly qualified, with 15 PhDs and 5 M.Sc. degrees, most of which are current graduate students from PPGBioinfo-UFMG or alumni. The selection of software used in this edition was focused on open-source, freemium*,* and low-priced tools. These tools were applied to email marketing, digital design, and the platform to manage the lectures. The lectures were made available on YouTube on live stream and later hosted in the CVBioinfo YouTube channel, with each lecture comprised of a unique video. During the event, we created an individual link for each talk (in favor of a single video containing all the lectures of that given day). After each talk, attendees were redirected to the next lecture. This was thought of to ease access to content and avoid program confusion by the attendees. Additionally, we provided interactive activities to engage and encourage the attendees to discuss the talks and ask questions to the speakers (Mariano et al., 2021).

## Results

**FIGURE 1:**
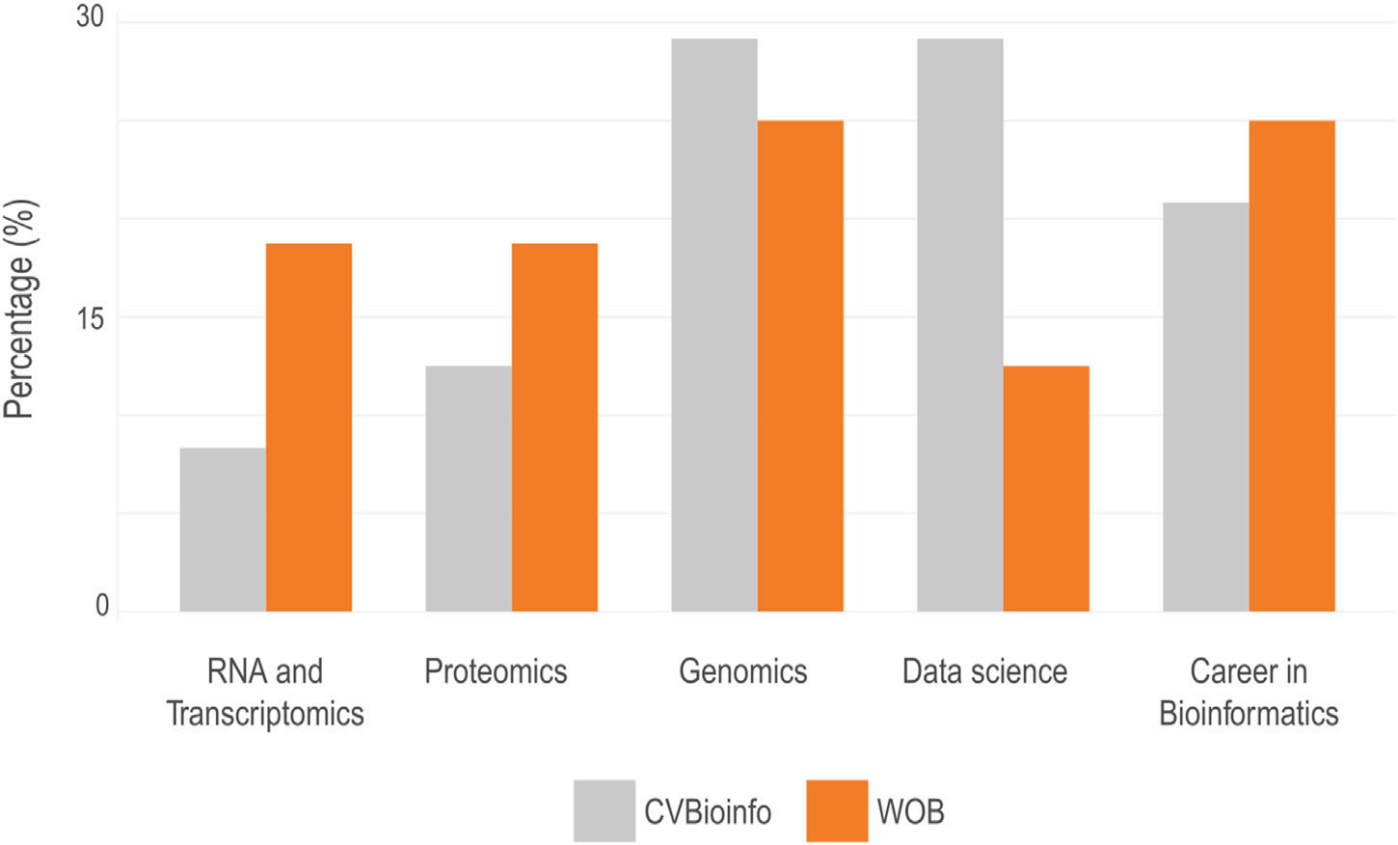
Proportion of scientific themes in bioinformatics from the schedule of lectures of CVBioinfo and WOB events. The main themes of the lectures were: **(A)** RNA and transcriptomics; **(B)** proteomics; **(C)** genomics; **(D)** data science; and **(E)** career in bioinformatics. Total attendees: WOB = 2,727; CVBioinfo = 221.

**FIGURE 2:**
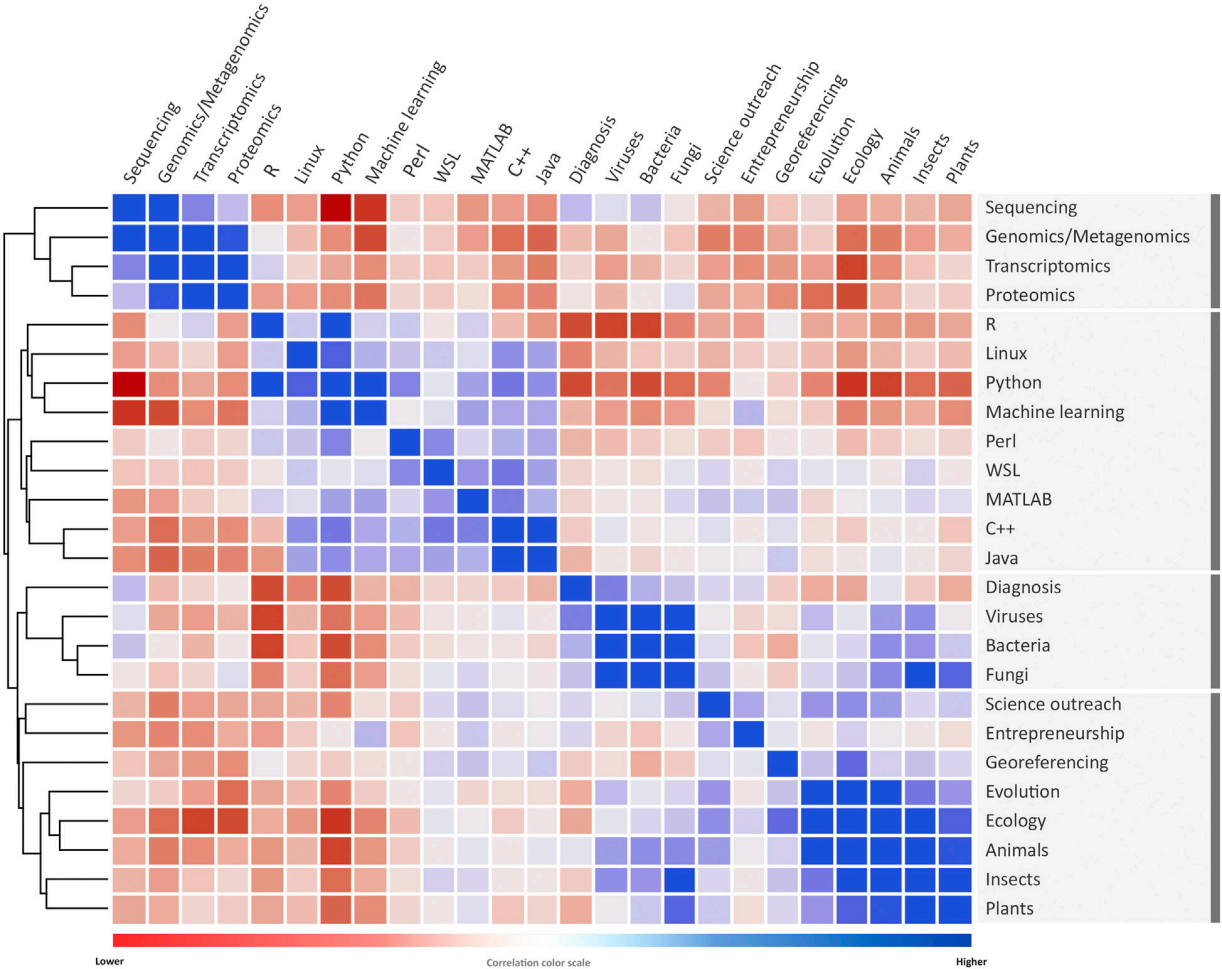
Correlation color matrix among most interest topics of attendees enrolled in the UFMG’s Online Workshop on Bioinformatics 2020 (WOB). From 2,727 students registered, we asked the question: “What are the five topics you are most interested in learning at WOB20?”. Moreover, we evaluated the correlation of topic pairs. In this visualization, blue cells represent a higher correlation between topics of interest, and red indicates a lower correlation (i.e., blue suggests that students interested in topic X–column–are also interested in topic Y–row). Some clusters were highlighted in rectangles with black borders and calculated using Euclidean distance. Figure generated using Orange Data Mining (https://orangedatamining.com/).

In order to illustrate the differences between organizing online and in-person events, we show here a brief comparison between the in-person event (CVBioinfo IV) that took place in January 2020 and the online event (WOB) that happened in November 2020 (Table 1). To officially apply to WOB, every attendee had to fill an application form to describe their subjects of interest in sciences, be it directly related to bioinformatics or not (Figures 1, 2).

**TABLE 1:** Comparison of structure in online and in-person editions.

Structure	WOB (Online)	CVBioinfo (in-person)
Reach	Worldwide	Local and visitors
Travel (Speakers)	No	Yes
Travel (Attendees)	No	Yes
Dependence on internet	Yes	No
Practical courses	No	Yes
Required infrastructure	Broadcast platform	Conference rooms with projector and enough seats for attendees and speakers

The overall distribution of subjects and specific fields within bioinformatics displayed in each event shifted between editions. For example, while genomics and proteomics kept their share of the program, RNA and transcriptomics-related lectures had a higher presence on the online event than in the previous in-person version. Moreover, entrepreneurial and career-oriented presentations for bioinformatics professionals increased, especially in the schedule of WOB. Nonetheless, a considerably smaller space in the schedule was dedicated to data science (Figure 1). We also evaluated the topics that incite the most interest among those enrolled. The list of topics included 25 topics related to computing and biology, which were defined empirically (Figure 2). For this question, each student could select up to five answers. The five topics of greatest interest to event attendees were: “Genomics/Metagenomics” (n = 1,439), “Sequencing” (n = 1,395), “Python” (n = 1,245), “R” (n = 1,014), and “Transcriptomics” (n = 931). Additionally, we correlated the most important topics of interest to identify which combination of subjects could arise the greatest interest from the attendees (Figure 2; Supplementary Figure S2). The heatmap retrieved three main groups composed of attendees with high interest in (i) computational topics (such as R, Python, Perl, and other programming languages); (ii) omics (such as genomics, transcriptomics, and proteomics); and (iii) exclusively biological themes. This information will be used, for example, to guide the choice of topics and speakers in future editions of the event. Another difference between the in-person and online events was the structure of the organizing committee. For the in-person events, the organizing committee had the support of a group of volunteers from graduate PPGBioinfo-UFMG and undergraduate students. Nevertheless, during WOB, the members of the Organizing Committee were sufficient to manage all the activities that include streaming the lectures and interact with the attendees on the chat, giving information about the event, answering questions, and collecting questions sent to the speakers.

**FIGURE 3:**
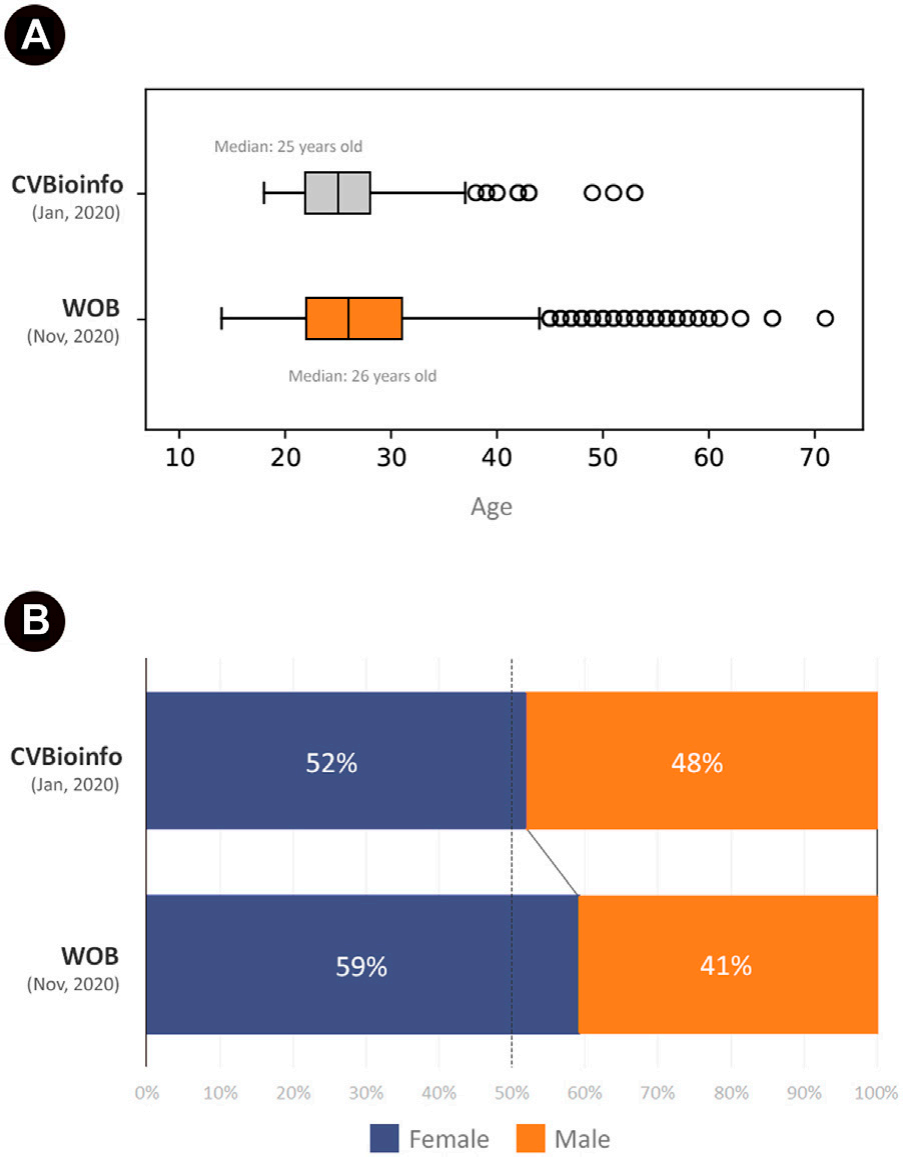
Attendees grouped by age and sex. Total attendees: WOB 2,727; CVBioinfo 221. **(A)** Quartile (0.25, 0.5, 0.75): 22, 26, and 31 (WOB); 22, 25, and 28 (CVBioinfo). Mode: 22 (WOB) and 25 (CVBioinfo). Mean: 27.46 (WOB) and 26.44 (CVBioinfo). Median: 26 (WOB) and 25 (CVBioinfo). Max value: 71 (WOB) and 53 (CVBioinfo). Min value: 14 (WOB) and 18 (CVBioinfo). **(B)** Male/Female proportion in CVBioinfo (upper stacked bar) and WOB (lower stacked bar). Conecting line between bars indicate change in values.

**FIGURE 4:**
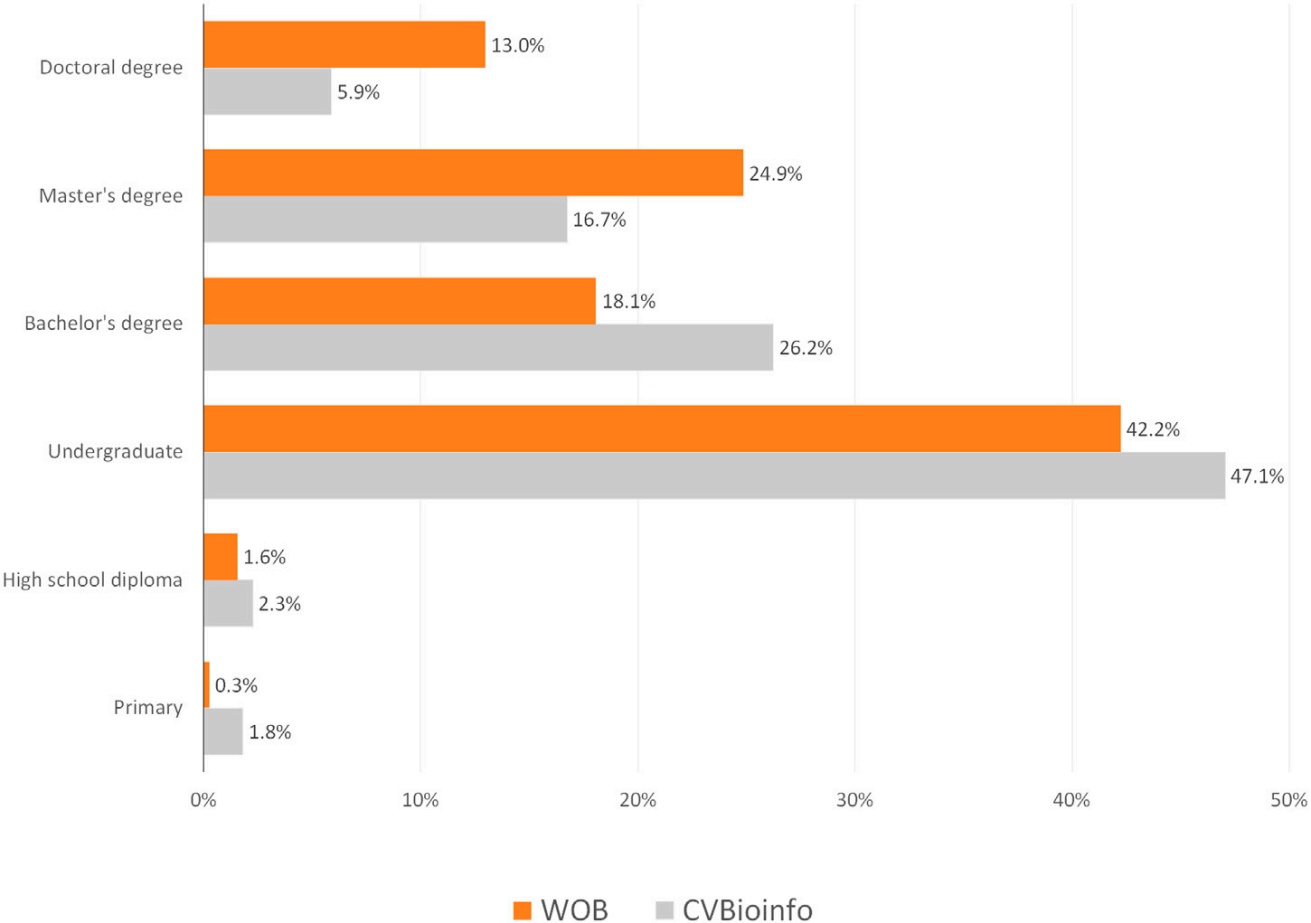
Attendees are grouped by education levels (percentual values). For M.Sc. and Ph.D. degrees, we considered completed or in progress. Total attendees: WOB = 2,727; CVBioinfo = 221.

**FIGURE 5:**
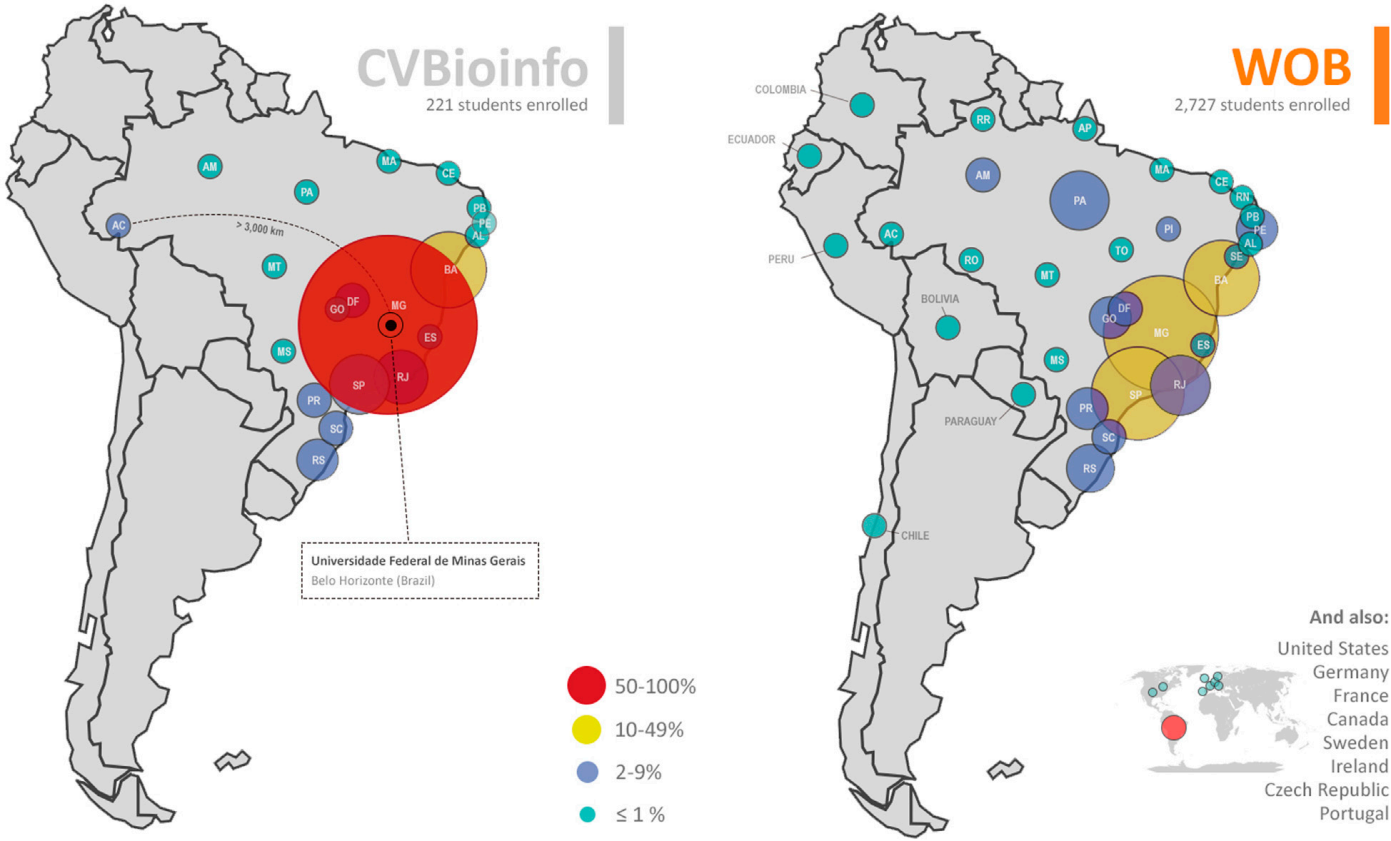
Comparison between the percentage of registrants in the face-to-face event called CVBioinfo 2020 **(left)** and in the online event, named WOB 2020 **(right)**. CVBioinfo - Summer Course of Bioinformatics 2020 (n = 221) and WOB–Workshop Online on Bioinformatics 2020 (n = 2,727). Registrations grouped by Brazilian states are shown on the bar plot in the center. Figure generated using Python (Plotly and pandas libraries), Microsoft Excel, and Adobe Photoshop.

The online event had a higher number of attendees enrolled (2,727) when compared to the in-person event (221). Most of the attendees in CVBioinfo were between 22 and 28 years old, while for WOB, at least half of attendees were between 22 and 31 years old (Figure 3A), representing a more diverse age group. The youngest attendee was 14 years old, while the oldest was 71 years old. Additionally, female attendees represented 52% of the CVBioinfo, against 48% of males, while in the online event, the number of female attendees increased to 59% (Figure 3B). The majority of the attendees were undergraduate students for both events (104 for CVBioinfo and 1,152 for WOB), and the number of attendees with bachelor degrees ranks as the second-highest for the CVBioinfo and third-highest for WOB (Figure 4); Figure 5 shows the countries in Latin America while comparing the percentages of attendees in the in-person event (CVBioinfo, on the left) and the online event (WOB, on the right), displaying a broader distribution of the origins of the attendees. Nevertheless, like the in-person event, most of the attendees were from the State of Minas Gerais (MG) in WOB. Other states with a high percentage of attendees are Sao Paulo (SP) and Bahia (BA). Furthermore, the online event allowed students from other countries to attend.

We requested feedback forms from the officially subscribed attendees of both events; however, each event form has probed for different information. CVBioinfo feedback aimed to assess the background knowledge of the attendees before the event, while WOB forms screened for the attendees’ interpretation of their own experience during the online edition. For the in-person setup, attendees described their skills on a scale ranging from zero to six, zero being no knowledge at all, and six related to an expert. From that group, mostly reported scores lower than four, that is, reported none or very basic level of understanding before the event. For example, 88.2% of all the feedbacks reported score lower than four in programming languages, 89.6% for GNU/Linux, 92.4% for molecular modeling, and 88.9% for bioinformatics in general. Meanwhile, only 7.7, 11.9, and 11.2% of the attendees that answered the feedback form declared to have no actual knowledge of genomics, proteomics, and transcriptomics, respectively. Both feedback tables are available in Supplementary Data (answers in Portuguese). Gathering a total of 143 replies (Supplementary Table S1), the answers collected by the CVBioinfo feedback form highlighted the knowledge gap previously displayed by the attendees of the in-person event.

For the online event, the focus of the feedback form was to connect with the quality of the experience for the attendees, especially considering this was the pilot trial for the online version of this event in bioinformatics. Gathering a total of 725 replies on the form (Supplementary Table S2), the attendees displayed high degrees of satisfaction with the adaptation of the event format for the online platforms (98.3%), scheduled program (99.0%), round conference tables (93.6%), and general organization (98%). Nonetheless, despite having been successful in its format, 42.1% of the attendees reported experiencing problems with internet connection during the Livestream, and 29.7% alleged to have difficulties remaining focused throughout the event.

To reinforce our values regarding inclusivity, besides the already mentioned LIBRAS interpreters, WOB partnered with a social project called Codigo X (available online at https://codigox.ong.br/), as a charity campaign. Codigo X is a non-governmental organization (NGO) that aims to introduce socially vulnerable girls in the technology field. As the online event had no participation fee, we encouraged the attendees to donate any value to support the activities of Codigo X. The approval feedback for both initiatives, language sign interpreters and the philanthropic campaign, was 99.2 and 97.8%, respectively.

## Discussion

Following a worldwide trend aimed to reimagine scientific conferences during the Sars-CoV-2 pandemic in 2020 (Hanaei et al., 2020; Jarvis et al., 2020), we compared the experiences of our online and in-person events for the popularization of Bioinformatics. Our attendee profile is slightly different between the events, with the average age of 25–26 years old, which is, in fact, our target audience: senior undergraduate students (Ristoff, 2014) probably interested in a career in bioinformatics. Female students correspond to more than half in both cases (52–59%) (IBGE, 2010). This proportion is equally displayed in our student team, where 57% of the organizers are women. Nonetheless, most lecturers were male for both CVBioinfo (69%) and WOB (62%). This reflects the changes in female access to college education in Brazil and abroad, which has increased since the 1980s to reflect the population proportion (Barros and Mourão, 2018). Furthermore, we can also correlate this proportion with the current preference for college majors in Brazil, also reviewed by Barros and Mourão (Barros and Mourão, 2018), in which men constitute the majority of the students in computational sciences, mathematics, and statistics majors. Although the data reviewed by these authors was relative to 2015–2017, approximately, female misrepresentation in some STEM fields continues to be a persistent obstacle reviewed elsewhere (Maarkert, 1996; Cheryan et al., 2017). According to (Moore, 1987), this gap seems to be present from the very beginning of the increase of female access to higher education in the 1960s and is still reflected in higher education staff throughout the late 1970s and early 1980s.

Besides a slight increase in female participation in the online event, the age range is also more diverse in this version, with more attendees over 40 years old. Although Ristoff described this cohort as a relevant part of undergraduate students, around 7.89% (Ristoff, 2014), in our experience, this cohort corresponds to graduated, employed professionals. Their participation might indicate a search for new methods and techniques already applied in their research since bioinformatics is now an indispensable part of different fields of expertise (ecology, epidemiology, pharmaceutics, and others). To contemplate this cohort, initially unexpected, we ought to bring more lectures on these topics of interest, in addition to our current panoramic perspective on bioinformatics, customized for a broader public. This category includes attendees in many different circumstances, for instance, people who have not completed their M.Sc. courses yet and some who did not attend graduate school, as well as people working in the industry, research centers, or even unemployed.

As the main positive aspect of our online event, the wide geographical distribution of the attendees was much broader than the in-person event. In CVBioinfo, we did not have more than five attendees from countries other than Brazil. On the other hand, the proportion of local attendees (from the metropolitan region of Belo Horizonte and the state of Minas Gerais as a whole) was much higher, likely due to transportation and accommodation expenses. In both events, neighboring states such as Bahia and Sao Paulo appeared with the highest number of attendees just after Minas Gerais. In contrast, the online event enabled the participation of individuals from the other Brazilian States that never attended all the previous editions of in-person events, such as from the Northern states (Amapa, Rondonia, and Roraima), from the Amazonian region, and also attendees from the states of Rio Grande do Norte, Piaul, and Sergipe, in the Northeastern region.

Besides Minas Gerais (the state where the event is situated), most of the other states displayed a significant proportional increase in participation, except for Acre, Alagoas, and Bahia. The Universidade Federal do Para (UFPA) possesses a highly active bioinformatics research group (available online at http://pctguama.org.br/), which can be correlated with their increased participation in the online event. Curiously enough, the Universidade Federal do Rio Grande do Norte (UFRN) possesses a Multidisciplinary Environment (available online at https://bioinfo.imd.ufrn.br/) and a Graduate Course in Bioinformatics. Nevertheless, it was quite surprising that the participation of this state in both the in-person and online events was still considerably low. This calls for a more collaborative effort for the scientific outreach in bioinformatics, similar to the RECOM Network (available online at http://www.recom-network.com/).

As exhibited in Figure 2, we can identify three major groups of interesting topics for the attendees. The first group was formed by Omics (Genomics, Transcriptomics, and Proteomics). The second group is formed by Computer Science, attendees interested in learning the GNU/Linux, programming language (R, Python, Perl, MatLab, C ++, Java), and machine learning while the third group showed greater diversity and correlation but mainly focused on Ecology (Viruses, Bacteria, Fungus, Animals, Insects, Plants, and Evolution). Curiously, cross-cutting themes such as scientific dissemination and entrepreneurship were correlated to this interest and not in a separate branch. The correlated areas displayed the need that each individual had according to their current training. Most of those enrolled were from areas focused on biological and biomedical sciences, and these students usually have experience in highly targeted research. By attending courses such as ours, which seek interdisciplinarity, the attendees can complement what they miss in their career formation and, thus, can search for complementary areas of interest.

In the in-person event (CVBioinfo), the attendees had the opportunity to participate in hands-on training. These training courses allow attendees to learn practice topics of interest to bioinformatics, complementing the topics covered in the lectures. In our experience, attendees tend to be very interested in hands-on training, and places used to be very competitive. In contrast, in this trial edition of WOB, we did not offer workshops and hands-on training, which are essential to most educators in Bioinformatics. A perspective to subsequent editions could be a model including hands-on training, short and interactive lectures as seen in (Abrahamsson and Dávila López, 2021), tools that involve the attendees as quizzes and sweepstakes, reinforcing the methods described by (Mariano et al., 2021), and activities to reach a greater regional diversity since the online event has worldwide coverage.

The increase in participation from states other than Minas Gerais resulted in more geographical diversity in our events, especially in the lectures. The student feedback was accessed through questionnaires that were sent to the attendees after the online event. Selected answers can be found in our Supplementary Data. While in the CVBioinfo event, the topics of most significant interest were related to the computing area (GNU/Linux, programming languages, and molecular modeling). This is probably due to that most of the attendees who had reported less knowledge were directly enrolled with these topics however, in WOB, the reported interest was much more spread. The topics of interest in WOB were focused on four main areas: Programming languages, Sequencing and Omics, Population and System Biology, and Machine Learning. Despite the great demand, WOB could not cover Programming language subjects and opened an excellent opportunity to improve the model hereafter. Overall, the attendees reported high satisfaction with the online event.

During all the events, there was a concern with interdisciplinarity. Thus, the lectures and courses did not focus only on computer science or biological sciences but, instead, they encompassed the intersection of these topics, so that the program was divided by theme and its applications, ranging from basic research to applications in industry. The invited professors and researchers belonged to different departments (Pharmacy, Biochemistry, Microbiology, Computer Science, Innovation, etc.), institutions (public and private), and companies (national and international). This was reflected in the satisfaction feedback with the diversity of available content. Therefore, this is a critical approach to be considered for the formation of the event schedule (Farina and Penof, 2020).

In-person CVBioinfo events had intervals between lectures, as well as dinner night aimed at promoting contact between attendees and CVBioinfo lecturers and PPGBioinfo professors. Those moments promoted interactions providing the opportunity for new partnerships to be established, as well as possibilities for attendees interested in attending PPGBioinfo as graduate students to find possible advisors. In contrast, the interaction between attendees and lecturers in the online event was limited to the live chat during the lectures and the contacts and social media the lecturers usually provided in their presentations. Nonetheless, we believe that the online event helped to promote our graduate program to a new public, which, in the case of graduate students from other institutions, might bring the possibility of scientific collaboration, for instance. To attract the attendees and overcome the loss of interaction, we promoted a gamification-based engagement strategy. This strategy consisted of grouping the attendees, based on their interest areas, into four distinct groups that competed against each other. An individual survey was sent to the attendees with questions related to the content of the lectures that occurred during the 3 days of the event. Attendees were then able to interact with other group members through exclusive discussion rooms. There was a high number of live messages among attendees during the competition, indicating that the strategy aided an increase in engagement and interaction between attendees (Mariano et al., 2021).

## Conclusion

Our results showed that the online event (WOB) we had offered increased access and was more inclusive when compared to our previous in-person events. Furthermore, organizing the online event was more straightforward compared to the in-person ones (CVBioinfo), especially regarding staff, since it was composed of a much smaller group of people than the in-person events, which, in turn, had larger groups with different responsibilities. Nevertheless, as the central negative aspect, our online event reduced the interactivity among participants. Moreover, problems with the internet connection (and the very access to the web) and difficulties to stay focused during the event were considered limiting factors. Therefore, we suggest that future educational events in bioinformatics should ensure that skills and information are more accessible, addressing the desires and expectations of the attendees. Thus, there is the perspective of creating thematic rooms or groups for the next events to be carried out by the Organizing Committee, aiming to increase the interaction between attendees and speakers. Most importantly, we suggest a hybrid format of in-person and online events.

## Data Availability

The original contributions presented in the study are included in the article/Supplementary Material, further inquiries can be directed to the corresponding author.
