# Supplementary material for: From In-Person to the Online World: Insights Into Organizing Events in Bioinformatics
Source: Front Bioinform. 2021 Sep 7;1:711463. doi: 10.3389/fbinf.2021.711463 (PMC9581051; doi:10.3389/fbinf.2021.711463)
Supplement: Supplementary file 2 [file DataSheet1.PDF]

Supplementary Material

1 Supplementary Figures and Tables

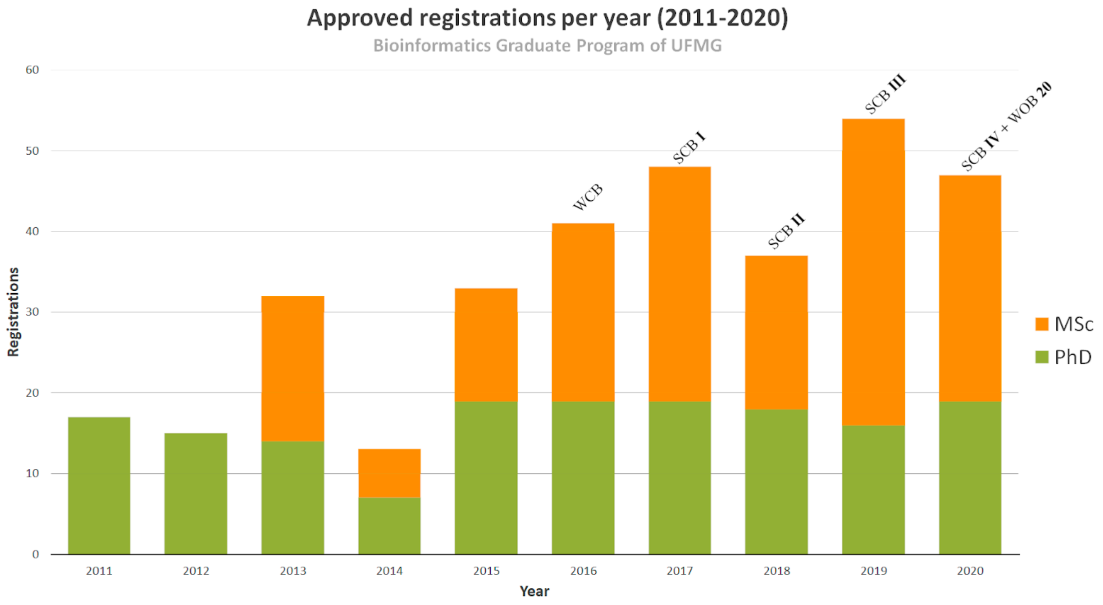

Supplementary Figure 1. Approved registrations for the selective process of the MSc and Ph.D. Bioinformatics Graduate Program of UFMG (2011-2020). The Master's degree program was created in 2013. WCB - Winter Course of Bioinformatics 2016. CVBioinfo I-IV indicates the face-to-face Summer Course of Bioinformatics occurred between 2017 and 2020, respectively. WOB20 indicates the Workshop Online on Bioinformatics occurred in November 2020. The list of registrations approved for the master and doctorate program was collected from the Graduate Program official website (available online at *http://www.pgbioinfo.icb.ufmg.br/*) using Python scripts and the libraries BeautifulSoup and requests. Data preprocessing was performed using regular expressions using the Python library re, Sublime Text tool, Google Sheets, and Microsoft Excel tool.

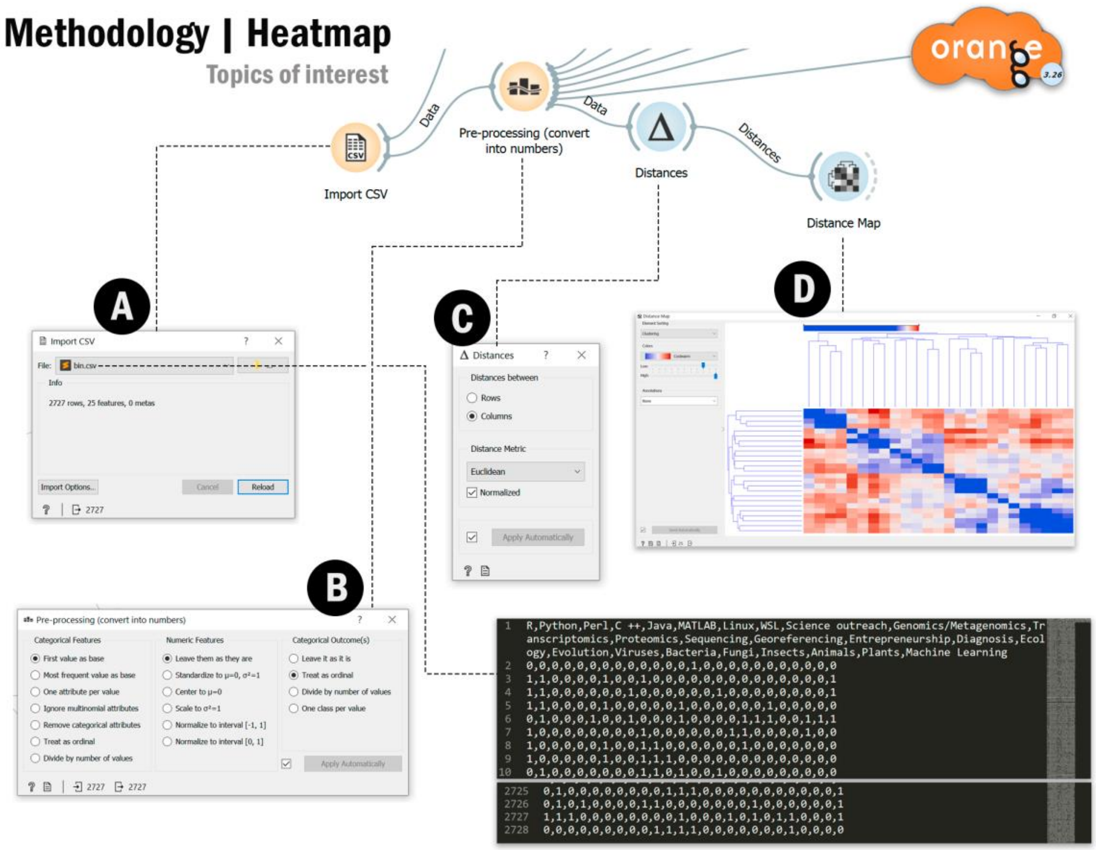

Supplementary Figure 2. Correlation among topics of interest of participants of the Workshop Online on Bioinformatics 2020 (WOB). We asked the participants "what are the five topics you are most interested in learning at WOB?". We then used the Orange data mining tool (available online at *https://orangedatamining.com/*) to generate this analysis; firstly, we asked the students to select five topics (from a list of 25 topics) that they had a major interest in. Then, we converted the answers into a binary matrix (as a CSV file), where each line receives 25 values separated by colons, which can be: (1) if the attendee had interest in the topic or (0) if they had no interest. Then, (A) we imported this data to Orange Data Mining, (B) pre-processing the data, (C) calculate the Euclidean distance among each answer, and (D) construct the heatmap. (D) Heatmap correlation between interest topics. And we evaluated the correlation of topic pairs. In this visualization, blue cells represent a higher correlation between topics of interest, and red indicates a lower correlation (*i.e.*, blue suggests that students interested in topic X - columns - are also interested in topic Y - rows).

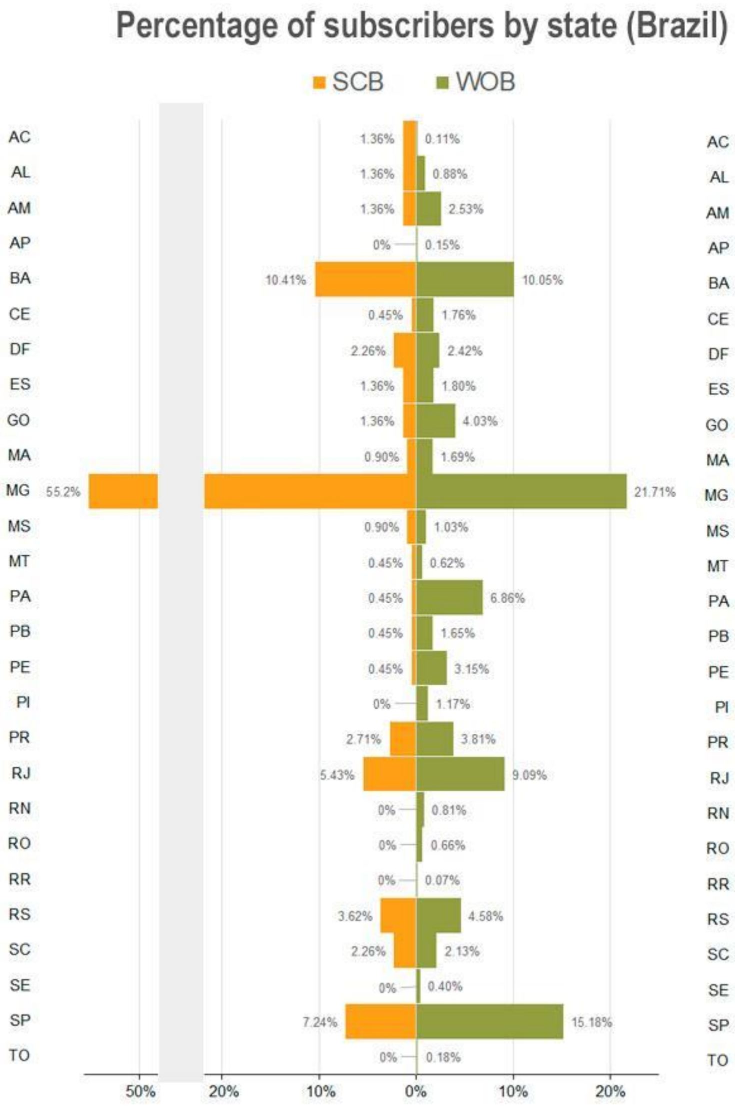

Supplementary Figure 3. Comparison between the percentage of registrants in the in-person event CVBioinfo (in the left, colored green, as "SCB") (n = 221) and in the online event, WOB (in the right, colored orange) (n = 2,727) in each State of Brazil.

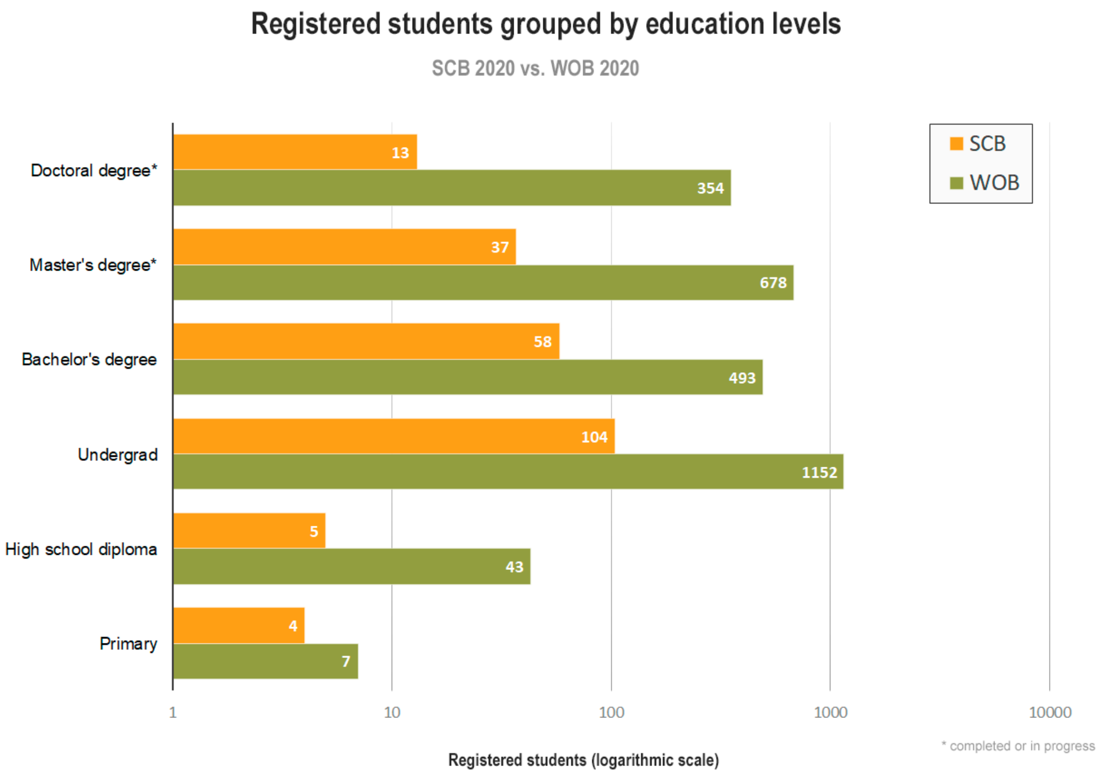

Supplementary Figure 4. Distribution of participants grouped by education levels (logarithmic scale). We considered M.Sc. and Ph.D. degrees as completed or in progress (*), for CVBioinfo (in the left, colored green, as "SCB") and in the online event, WOB (in the right, colored orange).
